# Understanding Risk and Protective Factors of Intimate Partner Violence: A Public Health and Social Advocacy Approach

**DOI:** 10.1007/s11121-026-01944-w

**Published:** 2026-07-01

**Authors:** Mangai Natarajan, Yasemin Irvin-Erickson, Brittany Suh, Susan Nembhard, Lindsay Smith, Yi-Fang Lu

**Affiliations:** 1 John Jay College of Criminal Justice, The City University of New York, New York, NY USA; 2 George Mason University, Fairfax, VA USA; 3 Old Dominion University, Norfolk, VA USA; 4 Graduate Center, The City University of New York, New York, NY USA; 5 University of Iowa, Iowa City, IA USA

**Keywords:** Intimate partner violence, Risk factors, Protective factors, Social advocacy, Public health, Mitigation, Prevention

## Abstract

Intimate partner violence (IPV) is a recurring social problem that has individual, interpersonal, community, and societal level consequences necessitating interdisciplinary knowledge and a holistic, systemic approach for prevention. Recent research on IPV indicates the need to identify the implications of the COVID-19 pandemic for IPV risk and subsequent consequences to inform mitigation solutions. Using a mixed-methods design, this rapid assessment research aims to address an existing gap by gathering primary data on the perceived risk and protective factors in addressing IPV through interviews, focus group discussions (*n* = 102), and surveys (*n* = 324) from a purposive sample of victim service providers (VSPs) in all US states, Washington, D.C., and five major inhabited territories (Puerto Rico, Guam, US Virgin Islands, American Samoa, Northern Mariana Islands). The study findings indicate that existing risk factors have increased, alongside the emergence of new ones at the individual, interpersonal, community, and societal levels. We also identified 35 proactive protection measures that VSPs concocted during the COVID-19 pandemic to mitigate IPV in their communities. Integrating insights from public health and social advocacy theoretical models, this study provides survivor-centered, community-level solutions to prevent IPV in both public emergencies and non-emergency situations. This research highlights the importance of recognizing the convergence of IPV risk factors during the COVID-19 pandemic, as factors such as the political climate, natural disasters, and health crises—including pandemics—can exacerbate existing risk factors, thereby underscoring the need for prevention efforts more than ever.

## Background

Intimate partner violence (IPV), which is commonly referred to as domestic violence (DV) or relationship violence, includes the physical, sexual, emotional, and financial abuse, among other controlling behaviors committed by a current or former intimate partner (Office for Victims of Crime [OVC], 2021; Office on Women’s Health [OWH], (n.d.). IPV is a significant public health concern that impacts the physical and mental health of millions of individuals throughout their lives, resulting in injury, disability, or death of victims in the most severe cases (Leemis et al., 2022). Moreover, IPV has negative consequences for communities and society, including increased public health costs, strain on social service agencies, and overall public safety. Studies on IPV trends in the United States (US) and worldwide have shown an alarming picture of abuse and violence during the COVID-19 pandemic, warranting greater attention. There remains a considerable gap in the literature on the risk and protective factors of IPV during the pandemic, particularly social support, which may provide important leverage for mitigating IPV and prevention in general and in future public health emergencies (Dutton & Goodman, 2005). Understanding the key risk and protective factors against IPV during the pandemic helps validate known risk factors and identify new ones that may exacerbate violence at home in the post-crisis/emergency period. The current study uses a social-ecological and social advocacy framework to address an existing gap by collecting data from victim service providers (VSPs) in the USA who have direct experience assisting IPV survivors. The aim is to improve our understanding of risk factors at the individual, interpersonal, community, and societal levels, and to explore protective factors, including neighborhood-level social support, that can help mitigate escalation, thereby informing social prevention strategies to effectively tackle IPV, a complex public health issue.

### What Do We Know About Pre-pandemic IPV Risk and Protective Factors?

The early, predominantly US-based studies identified several correlates of IPV risk factors at the individual and relationship levels, and a limited number of IPV risk and protective factors at the community and societal levels. Notably, recent meta-analyses grounded in ecological theoretical frameworks have demonstrated that some of the previously identified factors can exert stronger influences (see also Adames & Campbell, 2005; Ahrens et al., 2010; Benuto & Bennett, 2015; Goncalves & Matos, 2016; Natarajan, 2017).

Given the complex nature of IPV, a private yet social problem, studies using Bronfenbrenner’s (1979) Ecological Systems Theory (EST) have sought to understand the dynamic interactions among IPV risk factors within the immediate physical setting and across social environments, including the larger community and the social support system. Based on EST, a four-level social-ecological model of violence, and a review of 19 research studies, several factors have been identified that affect the risk of IPV perpetration at the individual, relationship, community, and society levels (CDC, 2024). Specifically, the CDC highlighted the association between IPV perpetration risk and the following factors: (1) biological and historical factors at the individual level, (2) the nature of close relationships in an individual’s social network, (3) characteristics of community settings, and (4) societal factors, such as social and cultural norms, policies, and regulations within a society.

Several individual and interpersonal risk factors for IPV have been identified in the literature. Additionally, studies conducted in the USA have highlighted community-related drivers of IPV, including higher levels of social disorder, violence, and poverty within communities, which are associated with an increased risk of IPV (see Benson et al., 2003; Blumenstein & Jasinski, 2015; Raghavan et al., 2006, 2009; Thulin et al., 2021). Furthermore, the CDC model (2024) has identified key societal factors contributing to IPV perpetration, including income inequality, weak domestic violence policies and laws, and cultural norms that endorse aggression toward others. On the other hand, neighborhood cohesion, coordination of resources among agencies within a community, and living in a community with access to safe, stable housing, health resources (including mental health), and economic support have been identified as protective factors against IPV perpetration. Spencer and colleagues (2019) examined risk markers for physical IPV victimization and IPV perpetration using Dutton’s nested ecological theory (1995), which emphasizes the role of the larger community and the social support system. Specifically, in their meta-analysis of 391 studies from multiple countries, including the US, they analyzed IPV victimization risk factors for men and women at the exosystem (social structures), microsystem (situational), and ontogenetic (individual) levels. Krebs and colleagues (2011) and Natarajan (2017) have further shown that physical IPV is more likely in relationships where multiple forms of IPV are perpetrated or where violence perpetration is bilateral.

### What Do We Know About IPV Risk and Protective Factors During the Pandemic?

Our review of the literature on IPV risks during the pandemic identified a limited number of original research studies conducted in the USA (Chalfin et al., 2021; Colton et al., 2023; Hicks et al., 2023; Rai et al., 2024), as well as studies from other countries, including Brazil (Correia et al., 2024) and Portugal (Cunha et al., 2024). Additionally, we found two evidence review studies from Argentina (Perez-Vincent et al., 2020) and Jordan (Alrawashdeh et al., 2024), as well as a multi-country rapid evidence overview (McNeil et al., 2023).

While one of the six research studies we identified utilized calls for service and other secondary data to understand IPV correlates during the pandemic (see Chalfin et al., 2021), the other five relied on original data collected from the general population (Colton et al., 2023; Hicks et al., 2023), shelter workers (Cunha et al., 2024), and populations that are high-risk for IPV, such as new mothers (see Correia et al., 2024) and immigrants (Rai et al., 2024). For instance, a study on the IPV experiences of immigrants in the USA found that the odds of IPV victimization were higher for immigrants with higher mental distress and limited family connections during the pandemic (Rai et al., 2024).

Some studies further signal that increased intense interpersonal stress, tension, and conflict in households due to unemployment/underemployment, food insecurity, and increased homeschooling were likely drivers of IPV during the pandemic (Alrawashdeh et al., 2024; Correia et al., 2024; Cunha et al., 2024; McNeil et al., 2023). Of over 1,000 individuals residing in 47 states and Washington, D.C., from Hicks and colleagues’ (2023) study, 56% of those who purchased a gun during the pandemic reported that they sometimes hit or punched their partner when compared to the 2 to 3% of non-gun owners and pre-pandemic gun purchasers/owners. Furthermore, several pandemic-related policies implemented by federal and local government entities were likely associated with an increased risk of IPV, due to increased time spent together in households (McNeil et al., 2023). Some pandemic studies from the USA and Argentina further showed that increased alcohol use became a problem to the extent of excess, leading to more IPV incidents (Chalfin et al., 2021; Colton et al., 2023; Perez-Vincent et al., 2020). These studies provide preliminary knowledge on the impact of the COVID-19 pandemic on IPV risk factors.

Ghoshal and colleagues (2023) indicated in their recent systematic review that risk factors outnumber protective factors in low and middle-income countries and emphasized that interventions to reduce risk factors and promote protective factors should consider the ecological level, as certain ecological-level factors are more amenable to intervention. This aligns with the US CDC’s (2024) ecological model, which highlights many risk factors but identifies few protective factors, suggesting the need to identify protective factors across ecological levels to inform the design of prevention interventions during public health emergencies and, more generally, beyond.

The above studies on IPV provide preliminary yet necessary knowledge on the impact of the COVID-19 pandemic on survivors and society. There is still a dire need for theory-informed and programmatic research into risk and protective factors for IPV/DV, which could shed light on various IPV prevention strategies, whether primary, secondary, or tertiary (see Capaldi et al., 2012; Evans et al., 2020; Hines & Douglas, 2018; Johnson, 2012;  Moreira & da Costa, 2020; Piquero et al., 2021; Shariati & Irvin–Erickson, 2025).

## Present Study

Using the CDC’s (2024) four-level social-ecological model, based on Bronfenbrenner’s EST, our study is to examine the risk factors associated with intimate partner violence (IPV) victimization and perpetration during the COVID-19 pandemic in the USA. We also incorporate Sullivan’s (2018) community-based advocacy model, which emphasizes an empowerment-based, survivor-centered, and action-oriented approach. This model acknowledges that IPV is influenced not only by individual experiences but also by broader relational, structural, and systemic factors. This aligns closely with the ecological framework, which helps to identify protective factors across multiple levels of influence. At the individual level, empowering practices such as safety planning, skill-building, information sharing, and supportive counseling enhance survivors’ coping capacities, autonomy, and emotional well-being. At the relationship level, advocacy strategies promote empathy, encouragement, and social support, thereby strengthening interpersonal connections and reducing isolation. At the community level, increasing access to resources, services, and community networks addresses structural barriers that affect survivors’ safety and recovery. Finally, at the societal level, Sullivan’s focus on systems and community change complements the social-ecological model, which considers broader social norms, institutional responses, and policy environments that influence IPV risk and prevention. While the social-ecological model outlines the various contexts where IPV risk arises, Sullivan’s advocacy model provides a practical approach to addressing these risks through survivor-centered interventions and systemic change. Integrating these two perspectives forms a strong foundation for examining both the determinants of IPV and the multi-level prevention and intervention strategies needed to support survivors’ safety and well-being. By merging the public health social-ecological framework with Sullivan’s service advocacy approach, our study aims to address gaps in the literature and answer the following research questions:

- **RQ1**: Which previously known individual-level, relationship-level, community-level, and societal-level IPV risk factors were more strongly associated with IPV risk during the COVID-19 pandemic?
- **RQ2**: What were the promising protective factors against IPV during the COVID-19 pandemic?

## Methodology

### Research Design

Given the descriptive and exploratory nature of our study, we chose Rapid Assessment Methodology (RAM), an intensive, mixed-methods design and an indispensable tool in emergency/rapid-response situations, such as the COVID-19 pandemic, to understand the multifaceted nature of IPV at the national level. RAM has been used in public health research to promote the integration of qualitative and quantitative approaches, enabling researchers and practitioners to capture the nuances of a specific environment to inform national-level policy recommendations (e.g., Natarajan, 2016; Palinkas et al., 2021).

### Data Collection

To answer our research questions, we developed an interview and a focus group discussion (FGD) protocol that included questions about the risk factors associated with IPV and protective factors that service providers perceived as helpful in mitigating IPV. Our survey, which was derived from existing literature, asked victim service providers (VSPs) questions related to the increase in the frequency of physical, sexual, emotional/verbal, and financial DV between March 2020 and March 2021 (in comparison to pre-pandemic patterns); individual-level risk factors (for both perpetrator and victim level); interpersonal, top five community-level and top three society-level characteristics that heightened IPV risk in general and during the pandemic.

We gathered both qualitative (interviews and FGDs) and quantitative (survey) data from VSPs who regularly interact with IPV survivors. Their insights are crucial for identifying IPV risk and protective factors, especially to meet the needs of survivors at the individual, community, and social levels. VSPs not only manage and mitigate individual-level risks but also identify and procure community resources to enhance survivors’ well-being. The protocol for our data collection was approved by the Institutional Review Board (IRB) of the first author’s academic institution.

#### Sampling Frame and Study Sample Selection

Lyon et al. (2011) estimated that there were about 1,900 domestic violence programs across the USA in 2011. We anticipate an increase in the number of programs over the years. Hence, using various data sources, including the websites of the National Coalition of Domestic Violence, the Office for Victims of Crime, Survivors.org, and targeted web searches of Family Justice Center (FJC) organizations, South Asian organization networks, and DV organizations in US territories, we developed a dataset. We identified 3,003 organizations that provided services to survivors of IPV across the 50 US states, Washington, D.C., and five US territories (i.e., American Samoa (AS), Guam (GU), Northern Mariana Islands (CNMI), Puerto Rico (PR), and Virgin Islands (VI)). In this initial search, we made a special effort to capture organizations that have targeted programs including immigrants, such as South Asian organizations, individuals with disabilities, ethnic/racial minorities, sexual and gender minorities, veterans, homicide co-victims, and organizations that serve rural survivors and survivors in US territories. While we believe this is not a complete inventory of service providers in the US, our compilation represents a concerted effort to develop a nationwide database of service providers (deposited on the ICPSR website: 209345).

Using this service provider’s US inventory, we identified 1,820 organizations (excluding law enforcement agencies, health institutions, and campus organizations in our sampling frame unless they have a specific DV unit/program) as our sampling frame. Of this total, 1,279 were identified as primarily serving IPV survivors, and 541 were serving IPV survivors as an umbrella organization along with other victims of crime and underserved populations.

#### Study Participants for the Interview, Focus Group Discussion, and Survey

For our semi-structured interviews and FGDs, first, we reached out to 1,279 organizations that primarily serve IPV survivors. Of those, 102 agreed to delegate participants for either an interview or an FGD via Zoom. Between June 2022 and November 2023, we conducted 73 interviews (each lasting 60 min) and 29 FGDs (each lasting 90 min) with informed consent.

For the survey, to enhance our study sample, we expanded our reach to include all 1,820 organizations that support both domestic violence victims and other types of victims, allowing for a comprehensive and scalable participant ecosystem. Of these, 423 organizations responded, accounting for 23% of the total. Notably, 77 of the 102 organizations we interviewed/participated in an FGD, also participated in our survey. For our survey data analysis, we included responses from 324 organizations whose representatives answered at least 90% of the survey (i.e., questions necessary to answer our research questions). Using Qualtrics, we fielded our survey between August and November 2023.

### Data Analysis and Results

RAM facilitated our navigation through the inductive–deductive process of scientific inquiry, with a robust emphasis on mixed-methods design and research analytics. The themes and questions in our interview and discussion protocol and in our survey questionnaire are informed by the same public health conceptual framework and the same sampling frame used to recruit our study participants. First, we processed the qualitative data derived from our interviews and FGDs through NVivo. While the questions were straightforward, the interviews were rich with subjective, detailed information. Four team members independently analyzed the transcripts assigned to them, after establishing intercoder reliability (Cronbach’s alpha = 0.863). To analyze our data from interviews and FGDs, we applied an inductive–deductive coding scheme to develop a preliminary list of themes on risk and protective factors aligned with our social-ecological conceptual framework, including individual, interpersonal, community, and societal levels, as well as Sullivan’s eight elements of service advocacy. We identified themes in NVivo and then used its crosstabulation and other analytical functions to create thematic summaries and tabulate how often participants mentioned each theme.

Drawing from existing literature on risk and protective factors, we created a survey questionnaire to address our research questions. We analyzed the quantitative data from the survey using SPSS. This analysis complements the qualitative data and allows us to accurately examine the distinct risk factors associated with each type of abuse.

### RQ#1: Perceived IPV Risk Factors During the COVID-19 Pandemic

#### Interview/FGD Results

Participants in our interviews and FGDs reported numerous key IPV risk factors during the pandemic across the individual, relationship, community, and societal levels. At the *individual level*, substance misuse, mental health disorders, and financial stress, especially when the perpetrator is the primary income earner, were reported as the strongest individual-level risk factors for experiencing or perpetrating IPV during the pandemic. One participant from a US territory (FGD 25) explained:

“It seems as if those now who are experiencing domestic violence are in a violent relationship, the partner has been taking either ice [Crystal Methamphetamine] or drinking. Some of them are mixing it with marijuana that’s laced with things and so having to deal with that … when she’s with him, he uses and takes it out on her. He either wants to have lots of sex with her, and then gets paranoid that she might be seeing someone.”

Other less commonly mentioned factors included immigration status, physical health, race, age, LGBTQIA + status, having a criminal record, fear of COVID-19, and stigma of IPV. At the *interpersonal/relationship level*, the study participants reported an increase in IPV due to staying at home, isolation from relatives and friends and other social networks, COVID-19-related stress, proximity to the abuser, controlling behavior of the perpetrator, and the presence of a weapon at home. A participant from a Southern state (Interview 43) explained:

“Well, isolation came right to their hands. We saw a lot of isolated people saying, ‘I haven’t heard from my sister, how can I get you guys to go see if she’s okay?’ … We saw that isolation and a lot of strangulation as well. We saw that they were needing us a lot more than before.”

Additionally, we noted that family stress, parenting stress, history of prior abusive relationships and assaults, financial stress, dealing with partner anger/hostility, taking care of pets, and relational stress all contributed to the increased likelihood of experiencing or perpetrating IPV during the pandemic. According to a participant from a rural area in a Western state (FGD 18):

“Every different type of abuse increased, it’s all about power and control with domestic violence. And, using that, whatever means to increase control. And I think the pandemic and the lockdown made people feel so out of control in so many ways that they exerted their power in other ways to try and gain that back.”

More narrowly, at the *interpersonal/relationship level,* perpetrator tactics were a significant risk factor associated with the COVID-19 pandemic. Study participants informed us that many of the perpetrators used various intensified controlling behaviors and coercion. One of the detrimental controlling behaviors noted was reproductive control, particularly over decisions about becoming pregnant and about continuing or terminating a pregnancy. The perpetrators monitored the survivors’ movements using digital devices and other surveillance, including GPS tracking. They subjected the survivors to multiple threats and the fear of immigration and deportation issues. While these factors may have been identified prior to the pandemic, as participants narrated, they were exacerbated during the pandemic, increasing individuals’ risk of experiencing IPV.

At the *community level*, our NVivo cross tabulation data of the interviews and FGDs indicated a long list of risk factors including: the lack of employment opportunities and resources, more children at home that were physically out of school, not having an internet connection, attitudes of the survivors including minimal support services and fear of reaching for help, lack of housing and lack of connection with friends and family, overall parental expectations around childcare, living in remote communities, immigration-related concerns, lack of therapeutic support, poverty, and living in an unstable or violent household.

At the *societal level*, changes in activities of law enforcement and the criminal legal system, immigration policies, political tensions concerning police brutality and targeting of ethnic/racial minorities, negative attitudes toward the national government, and COVID-19 restrictions were also found to have impacted IPV risk during the pandemic. According to the study participants, COVID-19-related disruption to public assistance and the criminal legal system was a risk factor that increased the likelihood of DV. As found, it was challenging to report and seek assistance for those with children. A participant from an organization serving the immigrant population in the Midwest (FGD 22) described a time when a client was reluctant to leave an abusive situation to seek help:

“She was really hesitant to leave the home because she had minor kids, babies, one is 18 months, the other one is three years [old]. And she said, ‘No this is my comfort zone. I don’t want to come out of the home.’ But the same night, the husband tried to put a criminal case on her. And she went to a correction center. She was there for two or three days. And later on, her case went from not okay to worst because she got so many cases piled up. Now she is dealing with custody evaluation for the kids. The abuser was trying to project in a such a way that she’s having anger issues and that there is no safety for the babies.”

Additionally, several participants described how the fear of COVID-19 affected survivors’ help-seeking for public assistance and VSP activities. For some service providers, like that of a participant from an organization in a Western state (Interview 55), help-seeking was thwarted by COVID-19: “The reports to child abuse services weren’t being made.” And another participant from a Southern organization (FGD 13) similarly stated: “There were probably fewer referrals to social services.” Regardless of limited reporting, a participant in the Northeast (FGD 1) explained the pandemic’s impact on service provisions: “The Department of Children and Family was not coming out. And even after things were opening up.” Between lack of clarity around service continuance and service limitations, one participant from a Midwestern organization (FGD 21) explained the harsh reality of the pandemic’s toll on survivors all around: “Many people thought all shelters were completely shut down; some of our clients actually had to quit their jobs because they had no one to help the kids with my remote learning.”

### Survey Results

#### Descriptive Findings on VSPs’ Perceptions of Top IPV Risk Factors

Survey responses allowed us to understand the VSPs’ perceptions on the top IPV risk factors at the individual and relationship levels during the pandemic and the most pressing community- and society-level drivers of DV in general. Table 1 (see overall column) shows that the top five individual-level risk factors for IPV perpetration during the first year of the COVID-19 pandemic were the perpetrator’s desire for power and control (70.4%), the perpetrator’s experience of economic distress (56.2%), the perpetrator’s heavy use of alcohol and drugs (48.1%), the perpetrator’s anger issues (44.1%), and the perpetrator’s prior history of physical abuse (40.7%). Also, the top five individual-level risk factors for DV victimization were having a jealous partner (61.1%), being a woman (59.0%), prior experience of IPV (56.5%), having dependents (48.1%), and unemployment (42.9%). All these prevailing risk factors were identified in prior literature.

**Table 1:** VSPs perceived individual-level top five IPV perpetration and victimization risk factors, and perceived increase in DV types during COVID-19 (*n* = 324)

	Overall	PA	SA	EA	FA
	%	Sig	Sig	Sig	Sig
**Risk factors for IPV perpetration**					
Desire for power and control	70.4	**0.002**	**0.026**	**0.003**	**0.002**
Economic distress	56.2	0.814	0.649	0.193	0.115
Heavy alcohol/drug use	48.1	0.484	0.822	0.409	0.434
Anger/hostility	44.1	0.127	**0.023**	**0.003**	0.216
Prior history of being physically abusive	40.7	**0.002**	0.909	0.056	0.821
Unemployment	30.2	1.000	0.462	1.000	0.628
Mental health issues	29.9	0.310	0.538	0.158	0.182
Belief in strict gender roles	22.8	0.071	0.503	0.124	0.427
Emotional dependence	19.1	1.000	0.318	1.000	0.671
Having few friends/being isolated	17.9	0.171	**0.027**	0.444	0.148
Prior history of being a victim of abuse	15.7	0.078	**0.031**	0.054	0.168
Depression	14.8	0.325	0.751	0.407	1.000
Low income	13	0.164	0.504	0.728	0.741
Low self-esteem	9.6	0.844	0.444	1.000	0.850
Involvement in aggressive/delinquent behavior in youth	4.3	**0.040**	1.000	0.154	0.589
Being an immigrant	2.8	0.504	1.000	0.723	0.736
Low academic attainment	2.5	0.457	1.000	0.723	0.735
**Risk factors for IPV victimization**					
Having a jealous and possessive partner	61.1	**0.032**	0.419	0.230	**0.030**
Being female	59.0	**0.007**	0.304	**0.012**	**0.003**
Prior history of intimate partner violence	56.5	0.196	0.650	**0.043**	0.222
Having dependents (children/older dependents)	48.1	0.162	0.214	0.061	0.577
Unemployment	42.9	0.558	0.821	1.000	0.910
Heavy alcohol/drug use	30.2	0.206	1.000	0.250	0.544
Being an immigrant	29.6	**0.007**	0.902	0.073	0.111
Witnessing or experiencing violence as a child	29.3	0.445	0.218	0.246	0.460
Being an ethnic/racial minority	25.0	0.283	0.090	**0.003**	**0.039**
Being transgender, nonbinary, or intersex	16.4	0.345	0.361	0.430	0.652
Being lesbian, gay, bisexual, queer, or asexual	12.7	0.295	0.735	0.112	**0.043**
Low academic achievement/education	7.1	0.256	1.000	1.000	1.000
Having a different ethnicity/race than a partner	4.0	0.773	1.000	0.555	0.398
High-risk sexual behavior	2.8	1.000	0.495	1.000	1.000
Not being married	2.8	0.070	0.742	0.166	0.308
Young age	2.8	1.000	0.495	1.000	0.084
Having a higher educational attainment than partner	1.2	0.613	0.147	0.603	0.329

Fisher’s exact test results (2-sided), statistically significant *p*-values in bold

*PA* physical abuse, *SA* sexual abuse, *EA* emotional and verbal abuse, *FA* financial abuse

Table 2 (see overall column) shows that the most frequently mentioned relationship risk factors for IPV during the pandemic were dominance and control in a relationship by one partner (85.2%), relationship conflicts (78.4%), unhealthy family relationships (73.5%), economic stress within the family (67.3%), and lack of involvement in conventional activities (56.5%). The top five community risk factors for DV during the pandemic were high rates of unemployment (59.9%), high rates of poverty and limited educational opportunities (56.2%), limited access to health care (51.5%), high rates of residential instability (50.3%), and limited access to services in communities (43.2%). At the societal level, cultural norms that support/normalize aggression (58.3%), weak enforcement of DV laws (55.6%), and societal income inequality and poverty (50.9%) were the most frequently mentioned factors for DV (see Table 3 overall column).

**Table 2:** VSPs perceived relations-level top five IPV perpetration risk factors and perceived increase in DV types  during COVID-19 (*n* = 324)

Relationship characteristics as risk factors for IPV	Overall	PA	SA	EA	FA
	%	Sig﻿	Sig	Sig	Sig
Dominance and control in the relationship by one partner	85.2	**0.001**	**0.017**	**0.001**	**0.001**
Relationship conflicts	78.4	**0.017**	0.132	**0.007**	0.078
Unhealthy family relationships and interactions	73.5	0.600	0.899	0.286	0.379
Family members experiencing economic stress	67.3	**0.018**	0.810	**1.000**	0.075
Lack of involvement in conventional activities	56.5	1.000	0.112	0.722	0.822
Parental substance misuse/criminality	46.0	0.198	**0.054**	0.289	0.219
History of experiencing poor parenting as a child	21.0	0.775	0.270	1.000	0.340
Parents’ lack of education	5.6	0.314	0.466	0.196	0.152

Fisher’s exact test results (2-sided), statistically significant *p*-values in bold

**Table 3:** VSPs perceived relations-level top five IPV perpetration risk factors during COVID-19 and perceived an increase in abuse types during COVID-19 (*n* = 324)

	Overall	PA	SA	EA	FA
	**%**	Sig	Sig	Sig	Sig
Community characteristics as risk factors					
High rates of unemployment	59.9	0.286	1.000	0.072	0.139
High rates of poverty/with limited educational opportunities	56.2	0.060	0.495	**0.001**	0.071
Minimal access to physical and mental health care	51.5	0.816	0.312	**0.045**	0.655
High rates of residential instability	50.3	0.294	0.261	0.157	0.738
Minimal access to social services	43.2	0.126	0.734	0.192	0.092
Low involvement among residents in intervening in DV	41.4	0.076	0.255	0.189	0.571
Easy access to drugs and alcohol	34.3	1.000	0.723	0.901	0.907
High rates of violence and crime	30.6	0.167	**0.050**	0.523	0.809
Easy access to guns	29.3	**0.041**	1.000	1.000	0.462
A lack of access to substance misuse treatment	24.4	0.346	0.360	0.784	0.436
Society characteristics as risk factors					
Cultural norms that normalize aggression toward others	58.3	0.906	1.000	1.000	0.910
Weak enforcement of DV laws	55.6	0.292	1.000	**0.044**	0.057
Societal income inequality/poverty	50.9	0.297	0.367	0.077	0.434
Traditional gender norms and gender inequality	29.6	0.799	0.902	0.699	0.627
Lack of respect for the rule of law and enforcement	21.9	0.483	0.892	0.479	0.501
Weak educational, economic, and social policies	20.4	0.389	0.578	1.000	1.000
Racial disparities	16.0	0.874	0.878	0.749	0.363
Loopholes in federal gun laws	11.1	0.711	0.474	0.138	1.000
Weak public health policies	6.2	0.087	0.162	0.327	0.171

Fisher’s exact test results (2-sided), statistically significant *p*-values in bold

*DV* domestic violence, *PA* physical abuse , *SA sexual abuse, EA* emotional and verbal abuse, *FA* financial abuse

#### Association Between the Increase in IPV Types and the Risk Factors During COVID-19

We further explored the bivariate association between our survey respondents’ perceptions of whether there was an increase in the frequency of different forms of DV during the first year of the pandemic and the top perceived IPV risk factors at the individual, relationship, community, and societal levels with Fisher’s exact tests (see Tables 1, 2, and 3).

*Physical Abuse:* perpetrators’ desire for control and involvement in aggressive delinquent behavior (*p* < 0.05), the victim being a woman *(p *< 0.01), immigrant status of victims (*p* < 0.01), having a jealous partner (*p* < 0.05), dominance and control in the relationship by one partner (*p* < 0.001), family members experiencing economic stress (*p* < 0.05), relationship conflicts (*p* < 0.05), and easy access to guns in communities (*p* < 0.05) were found to be associated with the VSPs’ perceived increase in physical abuse.

*Sexual Abuse*: Perpetrators’ anger and hostility issues (*p* < 0.05), perpetrators’ desire for control (*p* < 0.05), perpetrators’ isolation from others (*p* < 0.05), perpetrators having a history of being a victim of abuse (*p* < 0.05), dominance and control in the relationship by one partner (*p* < 0.05), parental substance misuse/criminality history (*p* < 0.05), and high rates of violence and crime in communities (*p* < 0.05) were found to be associated with the VSPs’ perceived increase in sexual abuse.

*Verbal and Emotional Abuse*: Perpetrators’ anger/hostility issues (*p* < 0.01), perpetrators’ desire for power and control (*p* < 0.01), perpetrators having a history of being a victim of abuse (*p* < 0.05), the victim being a woman *(p* < 0.01), victims identifying with a racial/ethnic minority group (*p* < 0.01), victims having a history of prior IPV victimization, dominance and control in the relationship by one partner (*p* < 0.001), a family experiencing economic stress (*p* < 0.001), relationship conflicts (*p* < 0.01), high rates of poverty/limited educational opportunities in communities (*p* = 0.001), minimal access to physical and mental health care in communities (*p* < 0.05), and weak enforcement of DV laws (*p* < 0.05) were found to be associated with the VSPs’ perceived increase in verbal and emotional abuse.

*Financial Abuse:* Lastly, VSPs’ observation of an increase in financial abuse during the pandemic was related to their perceptions of top IPV risk factors during the pandemic: perpetrators’ desire for power and control (*p* < 0.01), the victim being a woman (*p* < 0.01), victims identifying with a racial/ethnic minority group (*p* < 0.05), victims having a jealous and possessive partner (*p* < 0.05), and dominance and control in the relationship by one partner (*p* < 0.001).

#### Logistic Regression Results

We conducted logistic regression analyses to identify significant correlates of increased IPV cases during COVID-19, as reported by VSPs who participated in our survey. For this analysis, the four outcome variables captured VSPs’ perceived increase in IPV subtypes (i.e., physical, sexual, emotional/verbal, and financial) during the first year of the pandemic compared to pre-pandemic patterns. The independent variables in these analyses were the individual-, relationship-, community-, and society-level key IPV risk factors identified by VSPs that were significantly associated with increases in IPV types, as determined in bivariate analyses.

The results of the logistic regression (see Table 4) showed that, among the variety of risk factors identified by our survey participants, which the existing literature has also previously identified, a few emerged to have a significant association with VSPs’ perceived increase in physical, emotional, and financial abuse during the first year of COVID-19. Specifically, there was a significant relationship between VSPs’ perceived increase in physical DV and the following top IPV risk factors: perpetrator’s history of being physically abusive (OR = 1.84, *p* < 0.05), the survivor being an immigrant (OR = 1.84, *p* < 0.05), and family members experiencing economic distress (OR = 1.69, *p* < 0.05). There was also a significant relationship between VSPs’ perceived increase in emotional abuse and the following IPV risk factors selected as top risk factors during the pandemic: perpetrator’s anger/hostility (OR = 1.82, *p* < 0.05), high levels of poverty (OR = 1.75, *p* < 0.05), and family members experiencing economic distress (OR = 2.14, *p* < 0.01). Furthermore, at the interpersonal level, there was a significant relationship between VSPs’ perceived increase in financial abuse and dominance or control exerted by one partner in a relationship (OR = 2.26, *p* < 0.05), and at the individual level, having a jealous/possessive partner (OR = 1.72, *p* < 0.05).

**Table 4:** Logistic regression on the risk correlates with an increase in IPV types during the first year of COVID-19 (*n* = 324)

Variables	PA	SA	EA	FA
	OR (95%CI)	OR (95%CI)	OR (95%CI)	OR (95%CI)
Perpetrator characteristics				
Anger/hostility	–	1.56 (.97–2.49)	1.82 (1.08–3.09)*	–
Desire for power and control	1.43 (.80–2.56)	1.33 (.75–2.35)	1.26 (.68–2.33)	1.38 (.80–2.39)
Having few friends/being isolated	–	.55 (.29–1.06)	–	–
Involvement in aggressive/delinquent behavior in youth	6.93 (.83–57.90)	–	–	–
Prior history of being physically abusive	1.84 (1.08–3.12)*	–	–	–
Prior history of being a victim of abuse	–	1.78 (.94–3.35)	2.13 (.97–4.67)	–
Victim characteristics				
Being female	1.23 (.73–2.08)	–	1.19 (.69–2.05)	1.44 (.89–2.35)
Being lesbian, gay, bisexual, queer, or asexual	–	–	–	2.05 (.96–4.40)
Being an immigrant	1.84 (1.02–3.30)*	–	–	–
Being an ethnic/racial minority	–	–	1.80 (.92–3.53)	1.55 (.88–2.75)
Having a jealous/possessive partner	1.39 (.81–2.37)	–	–	1.72 (1.03–2.87)*
Prior victim of IPV	–	–	1.20 (.70–2.07)	–
Relationship characteristics				
Dominance/control in the relationship by one partner	1.57 (.72–3.39)	1.66 (.77–3.60)	2.03 (.93–4.44)	2.26 (1.07–4.76)*
Family members experiencing economic stress	1.69 (1.00–2.86)*	–	2.14 (1.24–3.70)**	–
Parental substance misuse/criminality	–	1.39 (.87–2.23)	–	–
Relationship conflicts	1.17 (.62–2.21)	–	1.11 (.57–2.13)	–
Community characteristics				
Easy access to guns	1.37 (.78–2.43)	–	–	–
High rates of poverty/limited education opportunities	–	–	1.75 (1.03–2.95)*	–
High rates of violence and crimes	–	1.41 (.85–2.33)	–	–
Minimal access to physical and mental health care	–	–	1.36 (.81–2.29)	–
Societal characteristics				
Weak enforcement of DV laws	–	–	1.43 (.83–2.46)	–
Nagelkerke R^2^	.16	.09	.22	.12

*PA* physical abuse, *SA* sexual abuse, *EA* emotional and verbal abuse, *FA* financial abuse, *DV* domestic violence, *IPV* intimate partner violence, *OR* odds ratio, *CI* confidence interval

^*^p < 0.05; **p < 0.01

### RQ#2: Promising Perceived Protective Factors Against DV During the COVID-19 Pandemic

#### Interview/FGD Results

As mentioned earlier, using the inductive-deductive coding process, we inferred approximately 105 themes related to protective factors at the individual, interpersonal, community, and societal levels, with some overlap among them. Drawing on Sullivan’s eight service advocacy principles, we then identified 35 protective factors at the community and societal levels that service providers perceived as mitigating the impact of the COVID-19 pandemic (see Table 5). The detailed findings are described below.

**Table 5:** Protective factors and social advocacy recommendations to promote IPV survivor well-being

Provide information and safety planning	Build skills	Offer encouragement, empathy, and respect	Provide supportive counseling	Increase access to community resources	Increase social support and community connections	System change work
Ensure consistent check-ins with survivors	Help survivors find jobs and prepare resumes	Supportive empowerment of survivors	Form a welcoming environment	Provide safe and pet-friendly shelters with high-capacity	Increase public awareness campaigns	Better framing of DV as a public health issue and increased partnerships with the community to identify victims
Be a messenger and recognize victims’ interests when providing information	Provide continuous employment assistance	Trust-oriented support system for survivors	Focus on making survivors feel comfortable	Provide extended or improved housing support	Improve collaboration between local organizations, including the police, courts, housing agencies, mental health agencies, educational institutions, and faith-based and non-faith-based NGOs	Provide a culturally sensitive, inclusive, and fear-free environment for victims to seek help
Recognize the unique information needs of immigrant survivors, including undocumented survivors	Provide safe relationship education	Incorporate survival and empowerment stories while engaging with victims	Focus on building rapport and trust relationships	Increase access to basic needs resources, including culturally specific food options, clothing, transportation, health care, and childcare	Increase partnerships with local businesses	Make policies to accommodate restorative justice when needed
Provide creative and flexible safety planning during public health emergencies	Implement school initiatives	Provide conciliatory guidance on public health regulation protocols	Trauma-informed care	Increase access to therapy, counseling, & wellness support	Increase support for immigrant survivors, including undocumented survivors	Enhancing the response to survivors by the criminal justice system
Provide DV training for all who are likely to encounter survivors	Provide education to enhance parenting capacities and parent–child bonds	Provide flexible survivor support	Provide peer counseling and support groups	Increase access to financial help	Promote community connectedness	Improve legislative changes to existing laws

This list was created based on Sullivan’s (2018) advocacy framework. Providing information and safety planning are grouped in one category

*1. Providing Information Rights, Options, and Experience (Including Safety Planning).* IPV survivors’ lack of knowledge about existing services necessitates increased awareness and training on IPV among a variety of stakeholders who may encounter survivors. In public emergency settings, these efforts involve tailoring information delivery and safety planning to each survivor’s needs, paying increased attention to hard-to-reach groups, given the disproportionate impact of public emergencies on certain survivor groups, and consistently implementing innovative strategies to engage IPV clients in these settings.

*2. Building Skills*. Our findings highlighted the detrimental effects of the pandemic’s financial and social consequences on households and individuals. Through the VSPs and other community partner programs, community-based efforts can enhance activities that help survivors of IPV develop real-life skills, such as support for securing and maintaining employment, safe relationship education, and parenting support.

*3. Offering Encouragement, Empathy, and Respect*. Providing empathetic, empowering, and trust-oriented support opportunities can increase survivors’ responsiveness to the knowledge and resources provided by victim advocates.

*4. Supportive Counseling*. Considering the differing needs of DV survivors, a supportive and trauma-informed counseling experience that makes survivors from different backgrounds feel welcome is warranted.

*5. Increasing Access to Community Resources and Opportunities.* In public emergencies, community-based organizations and advocates are vital for sharing information about essential resources. This includes safe, pet-friendly shelters; alternative housing; culturally specific services; free transportation and caregiving for children and seniors; mental health support; substance misuse services; financial assistance; and employment opportunities, with eligibility details.

*6. Increasing Social Support and Community Connections*. Increasing social support and strengthening community ties are vital to empowering survivors during long-term public emergencies, such as those following natural disasters. This involves raising awareness about available support networks and improving collaboration among government, non-profit organizations, and businesses in the recovery process, especially for those affected by IPV.

*7. System Change Work.* Community-level programs’ efforts on system-level advocacy to address IPV as a public health issue, including raising awareness of IPV risk factors, offering culturally specific services for survivors, promoting justice (particularly during public emergencies), strengthening domestic violence laws, and increasing funding for IPV services, would facilitate changes in the system to prevent IPV.

## Survey Results

Of the 15 community-level protective factors that we enlisted, the top five protective factors selected by survey respondents (*n* = 324) were access to safe and stable housing (70.7%), access to financial assistance (62.0%), access to mental health services (46.9%), increased funding for victim services (40.4%), and increased employment opportunities. These factors complement the protective factors identified through our interviews and FGDs.

In sum, the use of RAM’s complementarity in gathering rich qualitative primary data through interviews, FGDs, and surveys to answer our research questions reflects methodological pluralism, an essential embodiment of RAM, thereby providing validity to this nationwide study of IPV during the COVID-19 pandemic. For instance, findings from both qualitative and quantitative data complement each other, confirming existing risk factors from a social-ecological perspective. However, our inductive–deductive approach to the grounded nature of qualitative data provided a nuanced understanding not only of the perceived risk factors but also of the protective factors rarely reported in literature. The way we interpret qualitative data through an advocacy approach, complemented by the survey, is a novel endeavor.

## Discussion: Intimate Partner Violence Prevention

Public health literature constantly emphasizes the need to advance knowledge and innovation around IPV prevention (see Colucci & Hassan, 2014), including three tiers of prevention strategies: primary prevention involves removing the underlying root causes, secondary prevention involves immediate responses to occurrences, and tertiary prevention consists of responding to the long-term effects of IPV (HRSA, 2023). According to Chamberlain (2008), however, the distinctive types of prevention are not always clear-cut, and the levels of prevention are not mutually exclusive. This study, using the public health’s social ecology perspective, identified risk factors at the individual, interpersonal, community, and societal levels that are not considered the root causes of IPV. With a service advocacy approach to prevention, our research findings illustrate secondary and tertiary prevention for IPV, particularly service providers’ interventions that empower survivors and enhance their well-being by responding to their immediate and long-term needs, thereby preventing escalation and repeat victimization. Addressing protective factors, such as the suggestions in the “build skills” column (see Table 5), emphasizes primary prevention.

### A Public Health Risk Factor Approach

The COVID-19 pandemic has drawn attention to prolonged adverse conditions that have disrupted home life and led to an escalation in conflicts, potentially resulting in abuse and violence. According to the ecological framework, domestic violence is an outcome of unhealthy interactions among individuals, relationships, communities, and societal factors (WHO, 2021). Reflecting on Bronfenbrenner’s (1979) EST framework, our study’s findings highlight diverse risk factors across the social-ecological levels. Specifically, our qualitative findings demonstrated that the pandemic has had a lasting impact on IPV, emphasizing the need for targeted prevention efforts in public health policy.

On the *individual level*, significant alcohol and drug use, low academic achievement/education, and unemployment are identified as risk indicators for both survivors and perpetrators. Controlling behaviors, traditionally characterized as interpersonal risk factors, have diversified and intensified in the context of the COVID-19 pandemic, including tactics such as digital surveillance through GPS tracking. This technological abuse is not new, but the way in which perpetrators use it to control has emerged as a significant risk factor. This is indeed quintessential new learning about IPV perpetrators’ tactics that should be considered when developing prevention measures. At the *community and societal levels*, a deficient childcare system, inadequate community legal services, limited employment and educational opportunities, immigration laws and policies, restricted access to physical and mental health care, lenient DV laws and criminal legal justice, and a complex political environment intensified the risk of IPV, and the harm of victimization warrants attention.

Our quantitative findings signaled that financial and social repercussions of the pandemic could have increased odds of IPV victimization/perpetration in general through increased financial and social hardships experienced by individuals and households, especially for individuals who are more vulnerable to IPV due to increased isolation with likely perpetrators (i.e., immigrant survivors, individuals with possessive partners, or partners with prior IPV perpetration histories). Both pre-existing and emerging factors that impact individuals, relationships, and the broader community and social fabric indicate that the absence of support during lockdown exacerbates and leads to the emergence of IPV, putting vulnerable individuals at increased risk of IPV. The emergence of COVID-19 has had a sustained impact on individuals’ physical and mental well-being, posing considerable challenges, including effects on IPV risk. A wider range of risk factors, at both the individual and interpersonal levels, can interact with various social environments, including the political climate, emergency health crises such as the COVID-19 pandemic, and natural disasters. These interactions can result in significant stress and hardships, which may lead to an increase in IPV necessitating community-level prevention efforts.

### Advocacy Approach to Mitigating IPV

In a review of 15 studies, Kim and Royle (2024) affirmed the inadequacy of a single intervention to effectively mitigate all forms of IPV victimization during the COVID-19 pandemic, thereby suggesting the necessity of multifaceted and multidisciplinary strategies to address IPV, both amid and beyond the pandemic. Using Sullivan’s advocacy model (2018) as a theory of change, we organized the protective factors into seven headings (see Table 5) to illustrate the importance of social advocacy in promoting and protecting vulnerable survivors from victimization (and repeated victimization). These protective factors were translated into 35 community-level advocacy policy prescriptions to better serve IPV survivors and prevent IPV, both in general and during public health crises. Before the COVID-19 pandemic began, IPV was already a growing concern with various forms of risks. During the COVID pandemic, they evolved as new threats emerged. In response to these complexities, it has become essential to prioritize access to safe housing and to develop effective safety plans. Ensuring that individuals have the necessary support and resources is crucial for fostering their well-being and empowering them to regain their independence in a safe environment.

This national-level primary data study has provided valuable insights into service providers’ perspectives on patterns of abuse and highlighted key risk factors. It also identifies VSPs’ evidence-based solutions as best practices and protective factors for overcoming the challenges of assisting survivors during the pandemic, thereby informing future service plans. It offers practical implications for understanding a severe crime problem and for addressing research gaps and potential policy implications. It serves as a rigorous precursor for future national-level research. While we were able to capture risk factors in the home environment, such as perpetrators’ tactics and COVID-19 pandemic restrictions that led to isolation, our study data did not help identify safety and security measures at home. It may be because the participants’ roles as service providers are community-based. Future research can assist with such a detailed analysis.

In sum, despite several scholarly studies using primary and secondary data sources, including systematic reviews on the prevalence of factors contributing to IPV during the pandemic, a research gap remains for policy. This research is one of the few studies that use primary data to compile a comprehensive list of proactive protective measures at the community level to address IPV during the COVID-19 pandemic. We harnessed insights from VSPs who interact directly with IPV survivors, thereby obtaining valuable firsthand perspectives on IPV risk and protective factors. The crucial role these VSPs play in assisting survivors of IPV, on a day-to-day basis, especially during the pandemic, when law enforcement responses were constrained, further underscores the significance of our research.

### Limitations of the Research

Although our study, based on purposive sampling, captured only a proportion of VSPs’ insights from the US states, Washington, D.C., and the territories, it may limit generalizability to a specific population that faces barriers to seeking help. The use of RAM, a rapidly growing public health methodology, addresses the limitations of purposive sampling by integrating structured qualitative and quantitative techniques, which enabled our national-level understanding of the contextual and timely impact of the COVID-19 pandemic on IPV. Additionally, although our study primarily focused on IPV, our analysis of protective factors and community- and society-level risk factors may also have broadly addressed DV, given differences in VSPs’ definitions of IPV and DV, as well as their use of these terms interchangeably. Hence, the findings of this study should be interpreted in light of its methodological limitations. First, we recognize that relying on service providers’ perceptions of changes in the incidence of IPV and its risk factors is an unconventional approach compared with studies that use direct victimization data or administrative, population-based epidemiological datasets. While this perception-based method has recall biases and does not aim to establish causal relationships, as noted by Leigh et al. (2023), frontline providers were uniquely positioned to observe shifts in survivor needs, patterns of service utilization, and emerging risk factors during a time of significant social disruption. Second, our interviews and FGDs focused exclusively on service providers serving IPV/DV victims, and the survey, including umbrella organizations, had limitations due to our chosen subset of organizations. Third, we were unable to collect data directly from IPV survivors due to challenges posed by the pandemic. These included reduced accessibility, safety concerns, technology limitations, and heightened coercive control experienced by survivors during lockdown periods. Additionally, standardized administrative or program-level data suitable for cross-organizational analysis were not consistently available, as agencies varied in their data collection practices, reporting systems, and record-keeping completeness. Hence, the findings should be regarded as exploratory and practice-informed rather than representative of national epidemiological trends. Despite these limitations, the study provides valuable insights into the various risk factors associated with IPV during an unprecedented public health crisis. Furthermore, it identifies an extended list of protective factors that have been underrepresented in the existing literature beyond the COVID-19 pandemic.

## Conclusion

Our study represents a timely endeavor that leverages insights from VSPs across the USA to analyze risk and protective factors of IPV at the individual, relationship, community, and societal levels during the COVID-19 pandemic. Their input has been essential in understanding not only the pandemic’s impact but also the longstanding challenge of preventing DV in general and IPV in particular. By mapping the convergence of IPV risk factors during the COVID-19 pandemic, our research highlights the importance of acknowledging how elements such as the political climate, natural disasters, and health crises, including pandemics, can exacerbate existing risk factors at both the individual and relationship levels, thereby warranting a reevaluation of prevention efforts. Incorporating public health and social advocacy frameworks can influence public policy by addressing not only the risk and protective factors of IPV but also the public health-informed individual, interpersonal, and social-level determinants of IPV in both emergency and non-emergency situations. Effective public policies promote public health and safety, leading to sustainable, long-term solutions for preventing IPV that extend beyond the COVID-19 pandemic.

## Data Availability

Data for this study are not available due to the need to protect the privacy of study participants.
